# The Treatment of Morphinomania

**Published:** 1901-08-17

**Authors:** 


### THE TREATMENT OF MORPHINOMANIA.

Few cases in practice are so difficult to deal with as those in which the patient has become the slave of the morphia habit, and it is to be feared that the attempts made to overcome this habit are not always made with knowledge or with due consideration for the feelings of the patient. Dr. Oscar Jennings[1] insists, very rightly, that the mere suppression of the habit of taking morphia such as can be assured when a patient is under restraint is no proper cure, and that unless the craving for the drug is removed the habit will of a certainty return when the patient regains his liberty and when the conditions which have led to the taking of the morphia again arise. In regard to this craving, one must differentiate, when it will be found that it occurs in two forms. Hypodermic injections of morphia seem to give energy and "go," and it is for this reason that the syringe is resorted to on the slightest pretext. It is, says Dr. Jennings, the exact equivalent of brandy nipping. Thus one form of craving is a desire for the purely stimulating effect of the syringe. This can with comparative ease be got over by the exercise of some self control, or by a moderate amount of compulsion, so long as morphia in some other form is substituted. But the craving for the morphia itself is another matter. The morphia *habitué* becomes so dependent upon the drug that, quite independently of the immediate reviving effect which results from the use of the syringe, he depends, not merely for happiness but for bodily comfort, upon the regular ingestion of a certain daily dose of morphia, the lack of which is the cause of the miserable wretchedness that accompanies the sudden suppression of the opium habit. One must bear in mind that morphia is a stimulant, and especially a cardiac stimulant, and that not a few of the depressing effects of the sudden cessation of its use are due to the enfeebled action of the circulatory apparatus which immediately results. Hence the necessity of administering some form of cardiac tonic or stimulant while the morphia is being reduced. Again, it will be observed that the morphia *habitué* suffers greatly from "hyperacidity of the stomach and organism generally." No doubt much of the sinking and distress which these patients complain of is primarily due to this sour condition of stomach, and although the morphinomaniac will fly to morphia for the mitigation of this as of every trouble, still there are other ways of relieving it, such as by the administration of alkaline stomachics. Hence the great utility of bicarbonate of soda in the treatment of the morphia habit. In fact, in treating this condition it is not sufficient merely to cut off the drug. One must do all in one's power to remove every cause of discomfort, and to protect from every cause of distress, knowing as we do that in these patients the craving which is the source of the mischief is not a mere vicious desire for an unnatural stimulation, but is often a very natural longing to take what experience has shown to be the shortest way of getting rid of distressing feelings of many kinds.

In the treatment then of a case of morphinomania the first thing we have to do, according to Dr. Oscar Jennings, is to simplify the case by stopping the use of all other stimulants—alcohol, cocaine, or whatever they may be—which the patient may be taking; and it is comparatively easy to do this, for the morphia is what the patient craves for, and he will give up everything for its sake. Having then insured that we have but one vice to deal with, the next thing is to get rid of the use of the syringe by substituting some less harmful mode of administration; and this again ought not to be inordinately difficult; the patient can be persuaded to give up his temporary joy if we will allow him the means of escaping the continual misery which deprivation, or even material diminution of his daily dose of morphia produces. Dr. Oscar Jennings strongly advises that the morphia given for this purpose should be administered by the rectum. Of course the amount given in this way will have to be larger, perhaps twice as large as that taken hypodermically. But that does not matter. The patient is weaned from the constant desire for the immediately stimulating effect of the syringe. When this is accomplished we must gradually reduce the dose; as quickly as possible, but at the same time as slowly as is necessary to effect a cure without producing distress; and with the object of warding off this distress, we must administer cardiac tonics such as sparteine or digitalis. In some cases nitro-glycerine is useful.

Then, in view of the almost constant presence of hyperacidity, bicarbonate of soda should be administered; and lastly one must remember that the hot-air bath—the Turkish bath followed by massage and the cold douche—is one of the best of sedatives, and perhaps also tends to eliminate some of the toxic material which tends to accumulate in the blood of these "acid" patients. At any rate the use of the hot-air bath is of great service not only during the process of reducing the morphia but also afterwards. It is very important that as the morphia is reduced the patient should be kept to a strictly moderate and as a general rule a non-alcoholic régime. The tendency is for these patients, as they recover, to eat too much, which again brings on hyperacidity, and this in turn leads to a renewal of the craving. To prevent this a simple life with a fair amount of exercise and the regular use of Turkish baths is to be advised. The great thing is to get rid not merely of the morphia but of the craving for it; and this cannot always be done by strength of will, by good resolutions, or even by subjection to restraint, unless attention be paid to the conditions which lead to the desire for the drug, and also to the discomforts which follow the cessation of its use.

[1] Lancet, Aug. 10.

